# Integrated Care for Older People: Theories and Practices: Linlin Hu, Ye-Fan Wang Glavin. Beijing: Peking Union Medical College Press, 2022, pp 206, ISBN: 978-7-5679-2014-9 (paperback)

**DOI:** 10.5334/ijic.7658

**Published:** 2023-06-06

**Authors:** Zhihan Liu

**Affiliations:** 1 School of Public Administration, Central South University, Changsha, Hunan 410075, China

**Keywords:** integrated care, health care, social care, older people, person-centered care, China

## Abstract

As the first Chinese monograph to explicitly connect the “*Yiyang Jiehe*” with “integrated care for older people”, this book presents readers with a thorough overview of integrated care for older people, from theoretical content, management tools, to practices in China and international learning.

It was with excitement and anticipation that I turned my eyes to this book called “Integrated Care for Older People: Theories and Practices”. As a professor of health policy and management and a long-time participant in teaching and research in the field of integrated care for older people in China, I have been looking for a specialized publication that systematically introduces the concept frameworks and methodologies of integrated care for older people, and the development of practices in both international and Chinese context. I knew that such a work would eventually appear, but indeed, I did not expect it to come so quickly and with considerable depth.

As we all know, rapid societal ageing has been a global concern in recent years. In order to meet the growing diverse, multi-level demand for healthy ageing, the United States, the United Kingdom, Australia, Japan and other developed countries have established relatively comprehensive and characteristic service delivery and funding systems for integrated care. Over the past two to three decades, scholars in these countries have also made substantial contributions to the development of theories and methodologies of integrated care. However, to this day, we can observe that there are still fewer voices in academia from developing countries. This results in a lack of sufficient knowledge and practical evidences for the implementation and evaluation of integrated care models for older people in resource-limited settings. How to build an integrated care system with socio-economic suitability for a densely populated developing country like China that is “getting old before rich” and features a strong culture of filial piety, not to mention the long-time divide between its health care and social care systems? What lessons and successes can we learn from the past 8 years of practices of “*Yiyang Jiehe*” (meaning integrated health and social care for older people in Chinese)? What are the opportunities and challenges ahead? Until this book came out, our answers to these questions have not been clear enough.

It was written by two lead authors in integrated care. Dr. Linlin Hu is a professor of health policy and management from Peking Union Medical College of China. She has many experiences in national health policy and system research, with special interests in continuous and integrated health care system development and study. Professor Ye-Fan Wang Glavin is a teacher, researcher, advisor and author, has led many national policy reforms and model demonstration initiatives in the US and China. Her work has been often used as reference to support value-driven integrated system of care development.

The book starts with an introduction on the background and meaning of integrated care for older people, and is divided into three sections - theoretical, international and practical. In the Theoretical Section, the editors reviewed the international mainstream conceptual frameworks and typical success models represented by the World Health Organization’s framework for the implementation of Integrated Care for Older People (ICOPE), and constructs a comprehensive theoretical framework based on this. What is more attractive to me is the review of the assessment tools for integrated care of older people that follows. Here, we can see clearly that the assessment tools used in China are undergoing a transition from single-dimensional to multi-dimensional, and from single-system applicability to cross-system applicability. Moreover, we can find the direction for future research and application from comparisons of the advantages and disadvantages of various tools.

The International Section introduces the integrated care systems for older people in the United States, Japan, the United Kingdom, and Australia, including the context, development process, collaborative care models, and assessment and payment mechanisms of each system. The Practical Section presents a total of nine exemplars, which are distributed in pilot cities of integrated care for older people across China, such as Beijing, Shenzhen, Jinhua, Qingdao, and Jiaxing. The selection of each exemplar is based on its distinguishing attributes, such as the Beijing Geriatric Hospital, which is the pioneer in China in providing intermediate care (IC) that links acute and post-acute care; Beijing Yanda Center, which provides institutional care; Beijing Fengtai District, which provides home and community-based care; and Beijing Xicheng District, which provides smart home-based elder care beds. The authors also include their research findings on integrated aged care practices based on different financing arrangements in Jinhua, Qingdao and Jiaxing.

Overall, this book has the following strengths: (1) It is the first Chinese monograph to explicitly connect the “*Yiyang Jiehe*” with “integrated care for older people”. This means that a stable connection has been established and identified between a practical term with Chinese characteristics and an academical term that is internationally recognized in the field of aged care. This is of great significance for a country like China with a population of nearly 300 million people aged 60 or above. (2) It presents readers with a thorough overview of integrated care for older people, from theoretical content, management tools, to international and Chinese practices. (3) It highlights the attention paid to a series of policy priorities and research hotspots, such as community-based care and smart home care. The primary audience and the main beneficiaries of this book are undoubtedly researchers and policymakers in the field of geriatric health care. Additionally, it can serve as a reference book for graduate and undergraduate students studying health policy and management, aged care, nursing, social work, and other related disciplines.

In summary, I think this book provides theoretical frameworks and practical references for stakeholders cross-disciplinary, cross-organization and cross-care setting to co-design and co-develop a person-centered and value-driven integrated system of care. In my view, the next possible directions for research include: Firstly, conducting more analysis and summarization on the development of integrated care systems in the context of developing countries or Asian cultures. Secondly, more attention is expected to be paid to less developed areas with high complexity in human and physical geography, such as Hunan, Guizhou, Yunnan and other central and western regions in China, in the selection of cases.

